# Using Adaptive Imaging Parameters to Improve PEGylated Ultrasmall Iron Oxide Nanoparticles‐Enhanced Magnetic Resonance Angiography

**DOI:** 10.1002/advs.202405719

**Published:** 2024-08-20

**Authors:** Cang Li, Shanshan Shan, Lei Chen, Mohammad Javad Afshari, Hongzhao Wang, Kuan Lu, Dandan Kou, Ning Wang, Yang Gao, Chunyi Liu, Jianfeng Zeng, Feng Liu, Mingyuan Gao

**Affiliations:** ^1^ Center for Molecular Imaging and Nuclear Medicine State Key Laboratory of Radiation Medicine and Protection School for Radiological and Interdisciplinary Sciences (RAD‐X) Collaborative Innovation Center of Radiation Medicine of Jiangsu Higher Education Institutions Soochow University Suzhou 215123 China; ^2^ School of Information Technology and Electrical Engineering The University of Queensland Brisbane Queensland 4072 Australia; ^3^ School of Computer Science and Engineering Central South University Changsha 410000 China

**Keywords:** adaptive sequence parameters, CE‐MRA, dynamic concentration, mathematical optimization model, PEGylated ultrasmall iron oxide nanoparticles

## Abstract

The PEGylated ultrasmall iron oxide nanoparticles (PUSIONPs) exhibit longer blood residence time and better biodegradability than conventional gadolinium‐based contrast agents (GBCAs), enabling prolonged acquisitions in contrast‐enhanced magnetic resonance angiography (CE‐MRA) applications. The image quality of CE‐MRA is dependent on the contrast agent concentration and the parameters of the pulse sequences. Here, a closed‐form mathematical model is demonstrated and validated to automatically optimize the concentration, echo time (TE), repetition time (TR) and flip angle (FA). The pharmacokinetic studies are performed to estimate the dynamic intravascular concentrations within 12 h postinjection, and the adaptive concentration‐dependent sequence parameters are determined to achieve optimal signal enhancement during a prolonged measurement window. The presented model is tested on phantom and in vivo rat images acquired from a 3T scanner. Imaging results demonstrate excellent agreement between experimental measurements and theoretical predictions, and the adaptive sequence parameters obtain better signal enhancement than the fixed ones. The low‐dose PUSIONPs (0.03 mmol kg^−1^ and 0.05 mmol kg^−1^) give a comparable signal intensity to the high‐dose one (0.10 mmol kg^−1^) within 2 h postinjection. The presented mathematical model provides guidance for the optimization of the concentration and sequence parameters in PUSIONPs‐enhanced MRA, and has great potential for further clinical translation.

## Graphical abstract

A closed‐form mathematical model is demonstrated and validated to automatically optimize the imaging parameters for PEGylated ultrasmall iron oxide nanoparticles (PUSIONPs)‐enhanced magnetic resonance angiography (MRA), and the adaptive sequence parameters obtain better signal enhancement than the fixed ones during the prolonged measurement window.

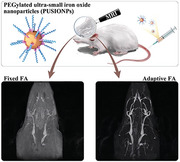

## 1 Introduction

Contrast‐enhanced magnetic resonance angiography (CE‐MRA) has achieved promising success in the early prevention and effective treatment of vascular‐related diseases. Long‐term CE‐MRA is apparently advantageous for the diagnosis and prognosis of various vascular diseases, such as stroke, myocardial infarction, and aortic dissection, by offering real‐time and dynamic vascular information.^[^ ^1^ ^]^ Gadolinium‐based contrast agents (GBCAs) are the most commonly used compounds in *T*_1_‐weighted CE‐MRA.^[^ ^2^ ^]^ However, recent studies warned that such GBCAs may cause nephrogenic system fibrosis and brain lesions in patients with kidney or liver conditions.^[^ ^3^ ^]^ In addition, the pharmacokinetic properties show that the concentration after the intravascular injection will drop rapidly due to the fast renal excretion, which may potentially require multiple contrast agent administrations and thus increase the risk of toxicity.^[^ ^4^ ^]^ Recently, ultrasmall superparamagnetic iron oxide nanoparticles (USIONPs, e.g., Fe_3_O_4_ nanoparticles) have shown great potential as a safe alternative to GBCAs because of their intrinsic nontoxic and biodegradable properties. USIONPs can be naturally metabolized into the hemoglobin and effectively cleared from patients’ bodies. More importantly, compared to GBCAs, USIONPs have longer blood residence time, thus enabling multiple imaging for monitoring the vascular conditions such as the location of embolization, the dynamic changes of vascular stenosis, and the postrecanalization treatment outcomes for the vascular embolism.^[^ ^5^ ^]^

The image quality of CE‐MRA largely relies on the contrast agent concentration and the parameters of the pulse sequences, such as the echo time (TE), repetition time (TR) and flip angle (FA).^[^ ^6^ ^]^ Since the concentration and the sequence parameters affect the *T*_1_‐weighted signal nonlinearly, making the full optimization of all factors quite complex. Previous studies used empirical observations to only optimize the contrast agent concentration at fixed pulse sequence parameters.^[^ ^7^ ^]^ Whereas it was only specific to the GBCAs used in the studies and may not produce the global optima for other sequence parameter combinations. To overcome these drawbacks, Reeder et al. developed mathematical models to automatically optimize the contrast agent concentration and parameters for the spoiled gradient echo sequence.^[^ ^8^ ^]^ Imaging results have shown that these mathematical models can successfully predict the optimal concentration and sequence parameters for various contrast agent types, e.g., gadobenate, gadoteridol, and ferumoxytol. However, the proposed mathematical models were only evaluated on phantoms or contrast agent‐filled catheters and may cause suboptimal results in more physiological environments, particularly for CE‐MRA.^[^ ^9^ ^]^ Besides, although the long blood half‐lives of USIONPs make it feasible to perform the multiple imaging in a prolonged measurement window, the intravascular concentration will change dynamically after the injection, which may require adaptive concentration‐dependent sequence parameters to achieve the optimal signal enhancement during the multiple imaging process. To the best of our knowledge, this effect has not been investigated in the present mathematical optimization studies.

In this work, we developed and investigated a mathematical model to automatically optimize the imaging parameters for PEGylated ultrasmall iron oxide nanoparticles (PUSIONPs)‐enhanced MRA using the 3D fast low‐angle shoot (3D FLASH) sequence and the fast spin echo (FSE) sequence. The pharmacokinetic studies were conducted to estimate the intravascular concentration within 12 h after the injection. The estimated dynamic concentration was incorporated into the presented mathematical model to determine the adaptive sequence parameters and to obtain the optimal signal enhancement in a prolonged acquisition window. The imaging performance and blood residence time were compared between the GBCAs and PUSIONPs. The phantom and in vivo rat CE‐MRA experiments were performed on a 3T animal scanner to validate the mathematical model.

## 2 Results

### 2.1 Relaxivities of the Fe_3_O_4_ Nanoparticles and Gd‐DTPA

**Figure 1:**
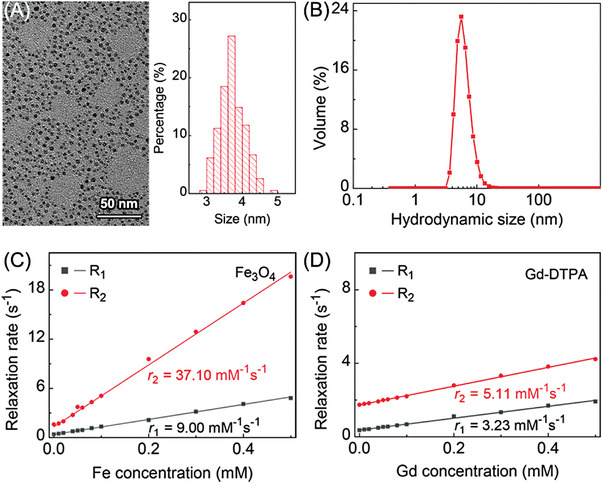
A) The transmission electron microscope (TEM) and particle size statistics of hydrophilic Fe_3_O_4_ nanoparticles; B) The hydrodynamic size of Fe_3_O_4_ nanoparticles; relaxivity values (*r*_1_ and *r*_2_) of C) Fe_3_O_4_ nanoparticles and D) Gd‐DTPA.

The core size and hydrodynamic size of the Fe_3_O_4_ nanoparticles were 3.72 ± 0.35 nm and 7 nm, respectively (**Figure** **1**A,B). The *r*_1_ and *r*_2_ values of the Fe_3_O_4_ nanoparticles were 9.00 and 37.10 mM^−1^ s^−1^, respectively, remarkably higher than the relaxivity values of the commercial Gd‐DTPA (*r*_1_ = 3.23 mM^−1^ s^−1^ and *r*_2_ = 5.11 mM^−1^ s^−1^), as shown in Figure 1C,D. The root mean square error of the linear fitting was 0.05 (*r*_1_ for Fe_3_O_4_ nanoparticles), 0.04 (*r*_1_ for Gd‐DTPA), 0.29 (*r*_2_ for Fe_3_O_4_ nanoparticles), and 0.03 (*r*_2_ for Gd‐DTPA), respectively.

### 2.2 Phantom Results

**Figure 2:**
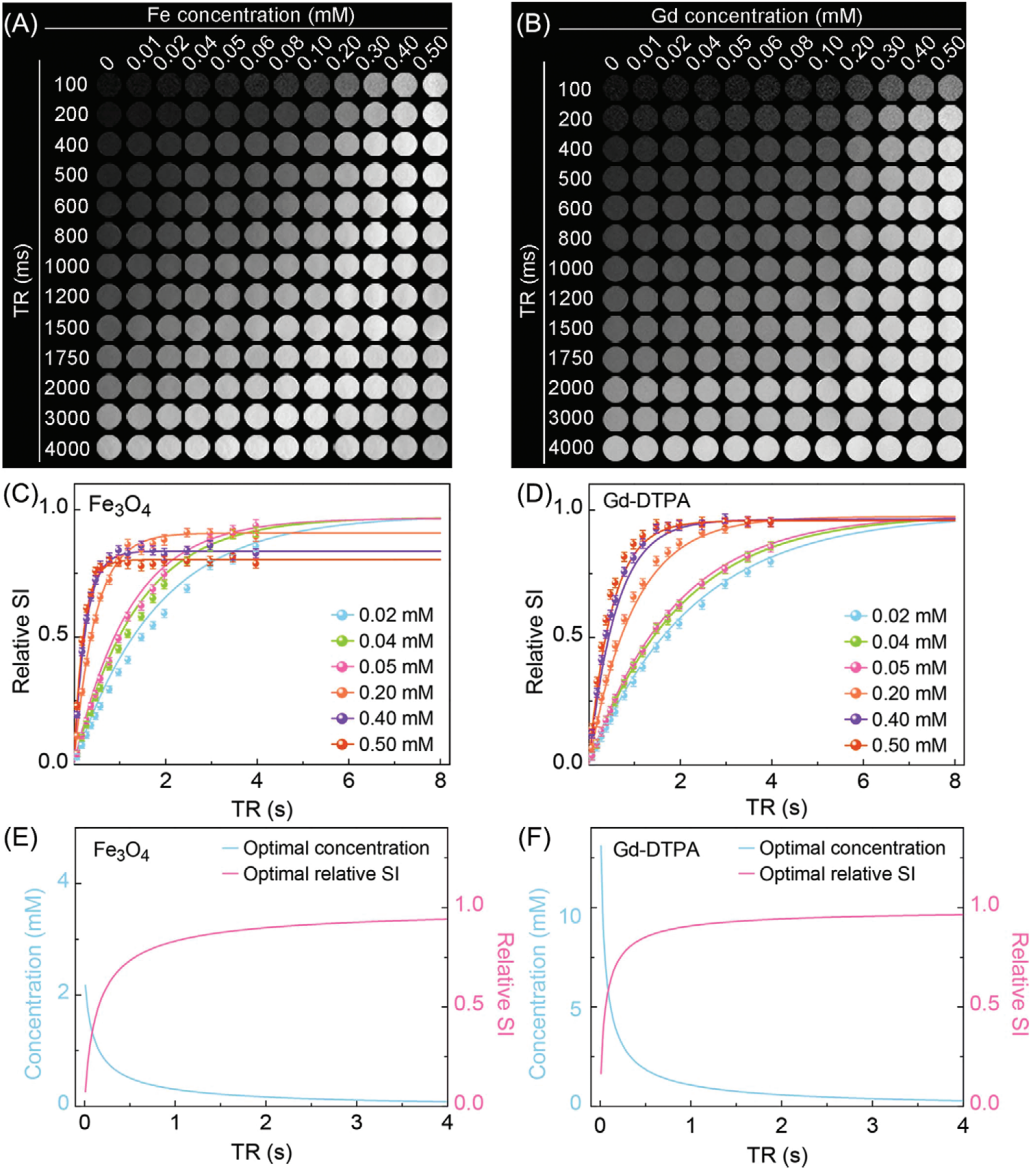
Phantom images acquired with the FSE sequence using A) Fe_3_O_4_ nanoparticles and B) Gd‐DTPA. Various concentrations (0–0.50 × 10^−3^ m) and TR values (100–8000 ms) were tested. The fitting of theoretically calculated (lines) and experimental (solid points) relative SI for (C) Fe_3_O_4_ nanoparticles (*R* ^2^ = 0.99) and D) Gd‐DTPA (*R* ^2^ = 0.96) at TE value of 11 ms. The optimal relative SI and concentration of E) Fe_3_O_4_ nanoparticles and F) Gd‐DTPA with respect to TR were calculated by the proposed mathematical model.

Phantom images of the Fe_3_O_4_ nanoparticles and Gd‐DTPA acquired with the FSE sequence using various concentrations (0–0.50 × 10^−3^ m) and TR values (100–8000 ms) were shown in **Figure** **2**A,B. When the applied TR was short, the signal intensity (SI) increased with the contrast agent concentration. For the case with long TR, the SI remained relatively stable with increasing concentrations. Relative SI was normalized with the *S*_0_ value. The theoretically calculated and experimental relative SI as a function of TR values (Figure 2C,D) were plotted using a range of concentrations (0.02 × 10^−3^ m, 0.04 × 10^−3^ m, 0.05 × 10^−3^ m, 0.20 × 10^−3^ m, 0.40 × 10^−3^ m and 0.50 × 10^−3^ m) at a minimal achievable TE value of 11 ms. A good consistency between experimental and theoretical data was shown for the Fe_3_O_4_ nanoparticles and Gd‐DTPA with *R* ^2^ values of 0.99 and 0.96, respectively. Figure 2E,F plot the calculated optimal concentration and relative SI with respective to TR values. The optimal concentration decreases with increasing TR values, while the relative SI peaked and then plateaued as TR increased.

**Figure 3:**
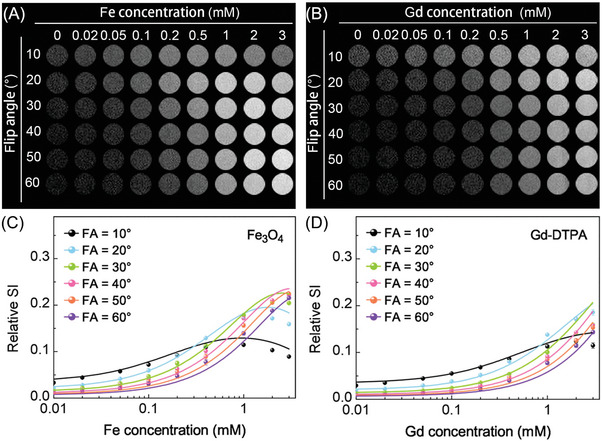
Phantom images acquired from the 3D FLASH sequence using A) Fe_3_O_4_ nanoparticles and B) Gd‐DTPA with TR = 10 ms, various concentrations (0–3 × 10^−3^ m) and FA values (10–60°). The fitting of theoretically calculated (solid lines) and experimental (points) relative SI against concentrations with various FA values (10–60°) for C) Fe_3_O_4_ nanoparticles (*R* ^2^ = 0.97) and D) Gd‐DTPA (*R* ^2^ = 0.96).

Phantom images of the Fe_3_O_4_ nanoparticles and Gd‐DTPA acquired with the 3D FLASH sequence among various FA values (10–60°) and concentrations (0‐3 × 10^−3^ m) were shown in **Figure** **3**A,B. The fitting of theoretically calculated and experimental relative SI against various concentrations for a range of FA values (10–60°) and TR value = 10 ms was plotted in Figure 3C,D. A good agreement between the theoretically calculated and experimental relative SI was demonstrated in these graphs with *R* ^2^ = 0.97 (Fe_3_O_4_ nanoparticles) and *R* ^2^ = 0.96 (Gd‐DTPA). It was also noticeable that the relative SI increased with increasing concentration of Gd‐DTPA; whereas in the case of Fe_3_O_4_ nanoparticles, the relative SI peaked and then dropped gradually as the concentration increased, indicating that the inherent relaxation properties of distinct contrast agents may lead to the different optimal concentration, under the same combinations of TR, TE and FA values.

### 2.3 In Vivo Rat Results

**Figure 4:**
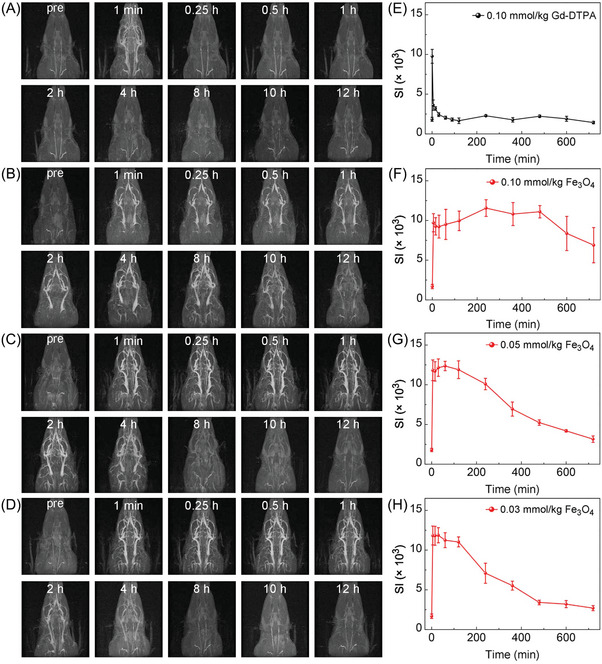
The 3D maximum intensity projection (MIP) reconstructions of rat CE‐MRA (TR = 10 ms, TE = 4 ms, FA = 30°) images within 12 h after the injection of A) 0.10 mmol kg^−1^ Gd‐DTPA and B–D) 0.10, 0.05, and 0.03 mmol kg^−1^ Fe_3_O_4_ nanoparticles, respectively. The SI values of the jugular vein over time for the injection of E) 0.10 mmol kg^−1^ Gd‐DTPA and F–H) 0.10, 0.05, and 0.03 mmol kg^−1^ Fe_3_O_4_ nanoparticles, respectively.

The CE‐MRA images of rats with Gd and iron doses of 0.10 mmol kg^−1^ were compared in **Figure** **4**A,B. The blood vessels became vague after 1 min in Gd‐DTPA‐enhanced MRA images (Figure 4A); however, these vessels were still clearly visible in Fe_3_O_4_ nanoparticles‐enhanced images (Figure 4B) within 10 h. The jugular vein SI of CE‐MRA images over time was plotted in Figure 4E,F. The SI for the Gd‐DTPA dropped rapidly after the injection. However, the SI for the Fe_3_O_4_ nanoparticles kept stable within 10 h, suggesting that the Fe_3_O_4_ nanoparticles enabled longer blood residence time than the Gd‐DTPA. Figure 4C,D shows additional CE‐MRA images with iron doses of 0.05 and 0.03 mmol kg^−1^; the corresponding SI curves are shown in Figure 4G,H. The SI remains at the same level as the conventional first‐pass MRA signal within approximately 2 h and then starts to decline gradually afterwards. The intravascular concentrations of the Gd‐DTPA and Fe_3_O_4_ nanoparticles within 12 h were estimated by a standard two‐compartment pharmacokinetic model (see Equation S7 and Table S1, Supporting Information) and were plotted in Figure S1 (Supporting Information). The intravascular concentration dropped rapidly after injecting 0.10 mmol kg^−1^ Gd‐DTPA, while the concentration remained at a high level within 4 h for the same amount of injection of Fe_3_O_4_ nanoparticles.

**Figure 5:**
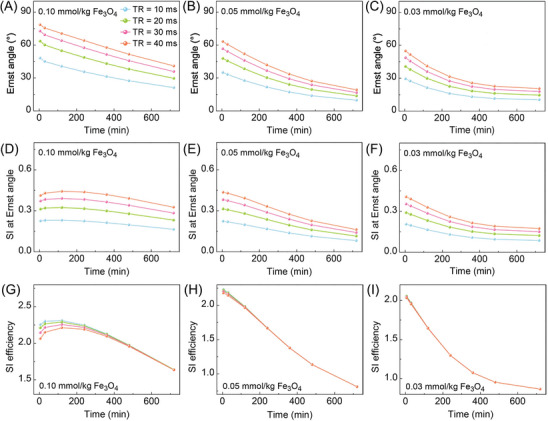
Theoretically calculated A–C) Ernst angle, D–F) SI at Ernst angle, and G–I) SI efficiency against the blood residence time given the combination of the estimated dynamic concentrations, TE = 4 ms and various TR values (10–40 ms). The calculations used the known *T*_1_ (1527 ms) and *T*_2_ (147 ms) blood relaxation times.

**Figure** **5** shows the Ernst angle, SI at Ernst angle and SI efficiency over time given the combination of the estimated dynamic concentrations, the TE and TR values. As Figure 5A–C indicates, the Ernst angle decreases with the circulation time. For the iron dose of 0.10 mmol kg^−1^, the SI at Ernst angle peaks at 100 min after the injection and then decreases gradually (Figure 5D). However, for low iron doses (0.05 and 0.03 mmol kg^−1^), the SI at Ernst angle decreased as the circulation time increased (Figure 5E,F). Figure 5G further reveals that the lowest TR value (10 ms) gives the best SI efficiency within 200 min after the injection of 0.10 mmol kg^−1^ Fe_3_O_4_ nanoparticles. The SI efficiency for different TR values stay almost the same after the injection of low‐dose Fe_3_O_4_ nanoparticles, as shown in Figure 5H,I.

**Figure 6:**
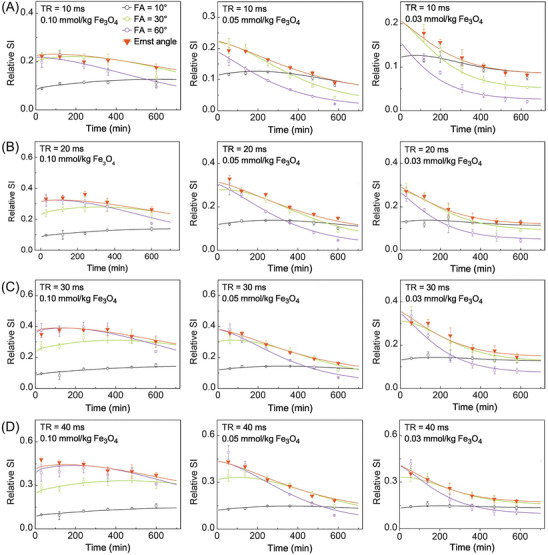
Theoretical (solid lines) and experimental (points) relative SI values of rat CE‐MRA images over time after the injection of 0.10, 0.05, and 0.03 mmol kg^−1^ Fe_3_O_4_ nanoparticles, with TE = 4 ms, various FA values (10–60°) and Ernst angle at A) TR = 10 ms, B) TR = 20 ms, C) TR = 30 ms, and D) TR = 40 ms.

To investigate the impact of adaptive sequence parameters on signal enhancement of different iron doses, three rats were administered with iron doses of 0.10, 0.05, and 0.03 mmol kg^−1^, respectively, and then scanned with the 3D FLASH sequence using various TR (10–40 ms), FA (10–60°), TE (4 ms), and calculated Ernst angle at 1, 15, 30, 60, 120, 240, 36, 480 and 720 min after the injection. As shown in **Figure** **6**, the theoretically calculated relative SI values are in good consistent with the experimentally measured values, which agrees with the results of Figures 2 and 3. Although the calculated Ernst angle is changed with the circulation time, it generates the highest relative SI values in comparison to other fixed FA values (10°, 30° and 60°), indicating that the adaptive Ernst angle can achieve the optimal signal enhancement.

**Figure 7:**
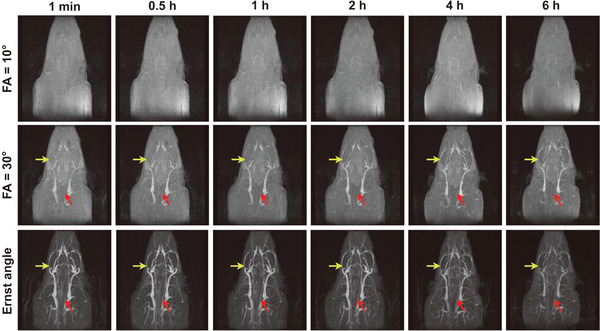
The 3D MIP reconstructions of rat CE‐MRA images within 6 h after the injection of 0.10 mmol kg^−1^ Fe_3_O_4_ nanoparticles acquired with the fixed FA values (10° and 30°, the top two rows) and the adaptive Ernst angle values (the third row), respectively at TR = 40 ms and TE = 4 ms. The red and yellow arrows denote the right common carotid artery and left posterior facial vein, respectively.

**Figure** **7** shows the rat CE‐MRA images with full thickness MIP reconstructions acquired with fixed FA values and adaptive Ernst angle values within 6 h after the injection of 0.10 mmol kg^−1^ Fe_3_O_4_ nanoparticles at TR = 40 ms. As indicated by red and yellow arrows, the right common carotid artery and left posterior facial vein are almost unseen in the images of the top two rows, while these arteries and veins can be clearly distinguished in the third‐row images, which demonstrates that the adaptive Ernst angle can provide better vascular enhancement than the fixed FA values during a prolonged measurement window. Figures S2–S4 (Supporting Information) show the rat CE‐MRA images with full thickness MIP reconstructions acquired with fixed FA values and adaptive Ernst angle values within 6 h after the injection of 0.10 mmol kg^−1^ Fe_3_O_4_ nanoparticles at TR = 10 ms, TR = 20 ms and TR = 30 ms, respectively, which are consistent with results in Figure 7. In addition, we also tested the performance of the proposed model on the thin MIP reconstructions, and the Figure S5 (Supporting Information) demonstrated that the adaptive Ernst angle can achieve better image enhancement than the fixed FA, which is consistent with full thickness MIP reconstructions in Figure 7.

## 3 Discussion

Due to the extracellular nature, the intravascular concentration of conventional GBCAs decreases rapidly after injection, and thus, GBCAs‐enhanced MRA typically applies first‐pass arterial phase imaging with limited acquisition time. In this work, the pharmacokinetic studies show that the intravascular concentration of PUSIONPs is maintained considerably longer than the commonly used GBCAs, enabling a sufficient time window for prolonged acquisitions. This advantage would facilitate diagnoses and treatments of arteriovenous malformations, which often require multiple imaging measurements pre‐ and post‐therapy.^[^ ^10^ ^]^ Stroke is the leading cause of deaths from cardiovascular diseases worldwide. Computed tomography (CT) has been widely used as the primary imaging in patients to rule out intracranial hemorrhage in the clinical practice. However, CT has a significantly low sensitivity to depict the acute ischemic stroke (AIS) with a sensitivity of only 12% in the first 3 h and 57–71% in the first 24 h after onsite symptoms, making it difficult to diagnose the AIS precisely and timely in such medical emergencies.^[^ ^11^ ^]^ On the contrary, magnetic resonance imaging (MRI) has greater sensitivity than CT for the AIS detection, which although is not as widely available as CT and has been utilized as a necessary follow‐up imaging for accurately diagnosing AIS and avoiding stroke mimics. There are two routinely used MRA techniques including time‐of‐flight MRA (TOF‐MRA) and CE‐MRA. CE‐MRA has shown better diagnostic accuracy compared with TOF‐MRA, particularly for identifying occlusion locations and visualizing collateral vascular. Thrombolytic drugs are clinically applied for the AIS treatment and such thrombolytic therapy is sometimes accompanied by intracranial hemorrhages, causing stroke‐related disabilities or even case fatality.^[^ ^12^ ^]^ Therefore, monitoring the dynamic changes of the vascular conditions especially at the embolization site is of great importance for evaluating the treatment outcomes. However, the current clinical GBCAs are incapable of achieving this goal with only one injection, a multiple injection is therefore required instead. It gives rise to a significant unmet clinical demand for evaluating the post‐therapy treatment for stroke patients. The current studies reveal that the long‐circulating time allows for multiple imaging measurements within 6 h in rats upon single dose of PUSIONPs, which will enable a serial and real‐time assessment of the thrombolytic therapy. In such medical emergency scenario, given the toxicity of the GBCAs and the complexity of multiple injections required, the single injection of PUSIONPs may become very attractive for evaluating the thrombolytic treatment through MRI.

Since the intravascular concentration of PUSIONPs changes dynamically during the long blood residence time, the adaptive concentration‐dependent sequence parameters are essentially required to obtain the optimal signal enhancement. Given the dynamic intravascular concentration, TR and TE values, the adaptive FA (Ernst angle) values were calculated with the presented model, and the imaging results have shown that it generated higher relative SI than the fixed FA values. It thus suggested that the presented mathematical optimization model could provide adaptive acquisition parameters to attain the best image quality for the CE‐MRA during a prolonged acquisition. Although the proposed mathematical optimization model has been only tested on PUSIONPs, it is readily applicable to other contrast agents with known information on relaxivity and concentration.

Various injected concentrations of PUSIONPs were tested for the in vivo rat CE‐MRA, and the results showed that the low‐dose contrast agents (e.g., 0.03 and 0.05 mmol kg^−1^) gave comparable SI to the high‐dose one (0.10 mmol kg^−1^) within 2 h after the intravenous injection, indicating that the high relaxivity and long blood half‐lives of PUSIONPs are beneficial for reducing the injected doses for the CE‐MRA applications. These experimental validations would also be helpful for guiding the contrast agent dosing protocols. Moreover, the maximal possible SI predicted for a given concentration, TR and TE combination, could be used to quantify the imaging performance of a contrast agent. As shown in the mathematical equations, the SI is increased monotonically with TR. However, when the TR is too long, the *T*_1_‐weighted effect will be weakened due to the longitudinal magnetization recovery. It may explain that the relative SI is almost the same when TR is larger than 4 s in Figure 1C,D. It is notable that increasing TR also increases the acquisition time, making it impractical for some fast CE‐MRA applications, e.g., catheter‐based interventions and interactive coronary MRA.^[^ ^13^ ^]^ Therefore, we define herein the SI efficiency that is monotonically increased with decreasing TR, leading to a trade‐off between SI and efficiency. Recently, MR acceleration techniques such as compressed sensing and parallel imaging have shown promise for the dynamic MRA.^[^ ^14^ ^]^ We will investigate the application of PUSIONPs‐enhanced MRA to MR acceleration problems to further reduce the acquisition time in the future.

The relaxivity of contrast agents is highly dependent on the physiological environment. Studies have shown that the longitudinal relaxivity of the contrast agent in blood is nonlinear at high concentrations.^[^ ^15^ ^]^ Furthermore, the transverse relaxivity would be impacted substantially in the presence of the endogenous erythrocytes, which may result in microscopic magnetic field inhomogeneities.^[^ ^16^ ^]^ The mathematical description of the relationship between relaxivity and the environment will be investigated and incorporated into the presented mathematical optimization model in the future to provide more precise predictions.

## 4 Conclusion

In this work, we proposed a mathematical model that incorporated adaptive concentration‐dependent sequence parameters for optimizing PUSIONPs‐enhanced MRA. Through both phantom and in vivo rat imaging studies, a good consistency between the experimental measurements and theoretical predictions was obtained. On top of that, with reference to conventional GBCAs, a longer acquisition window with a lower dose was enabled for PUSIONPs, which may open up a bright way to precisely diagnose cardiovascular diseases through PUSIONPs‐enhanced MRA. Beyond that, the current model may also be applicable for other types of contrast agents in blood pool imaging.

## 5 Experimental Section

### The Mathematical Optimization Model

The SI in the *T*_1_‐weighted FLASH sequence can be formulated as:^[^ ^17^ ^]^

(1) $$\mathrm{SI}=\frac{S_{0}\left(1-e^{-\mathrm{TR}\left(R_{10}+r_{1}C\right)}\right)\sin\left(\mathrm{FA}\right)}{1-\cos\left(\mathrm{FA}\right)e^{-\mathrm{TR}\left(R_{10}+r_{1}C\right)}}\;e^{-\mathrm{TE}\left(R_{20}+r_{2}C\right)}$$

where *S*_0_ represents the longitudinal magnetization at equilibrium and FA is the excitation flip angle. *R*_10_ and *R*_20_ are the longitudinal and transverse relaxation rates without the presence of contrast agents, and *C* is the contrast agent concentration. *r*_1_ and *r*_2_ denote the longitudinal and transverse relativities of the applied contrast agent, respectively. The aim of the optimization is to determine the values of TE, FA, and TR that maximize the SI.

(2) $$TE_{\mathrm{optimal}}=TE_{\min}$$

By taking the derivative of Equation (1) with respective to *FA*,  the optimal *FA* occurs at the well‐known “Ernst angle”:^[^ ^18^ ^]^

(3) $$FA_{\mathrm{optimal}}=\mathrm{co}s^{-1}\;\left(e^{-\mathrm{TR}\left(R_{10}+r_{1}C\right)}\right)$$

It is noted that $\cos\;\left(FA_{\mathrm{optimal}}\right)=e^{-\mathrm{TR}(R_{10}+r_{1}C)}$ and $\sin\left(FA_{\mathrm{optimal}}\right)={\left(1-e^{-2\mathrm{TR}(R_{10}+r_{1}C)}\right)}^{1/2}$. Inserting the optimized TE and FA to Equation (1) can yield the equation below:

(4) $$\mathrm{SI}=S_{0}\;\sqrt{\frac{1-e^{-\mathrm{TR}\left(R_{10}+r_{1}C\right)}}{1+e^{-\mathrm{TR}\left(R_{10}+r_{1}C\right)}}}{e^{-TE_{\min}}}^{\left(R_{20}+r_{2}C\right)}$$

By taking the derivative of lnSI with respect to the concentration *C*,  the *C*_optimal_ that maximizes the SI can be calculated as:

(5) $$C_{\mathrm{optimal}}=\frac{\ln\left(D+\sqrt{1+D^{2}}\right)}{r_{1}\mathrm{TR}}\;-\frac{R_{10}}{r_{1}}$$

where *D*  = (*r*_1_TR)/(2*r*_2_TE_min_)  represents the ratio of longitudinal recovery and transverse decay, and thenEquation (1) can be rewritten as:

(6) $$\mathrm{SI}=S_{0}\;\frac{D+\sqrt{1+D^{2}}-1}{D+\sqrt{1+D^{2}}+1}$$

Equation (6) can be used to determine the impact of TR on the maximal SI. Mathematically, as ∂SI/∂*D* > 0 and increasing TR will increase the SI. However, increasing TR will also increase the scan time. For some MRA applications such as real‐time catheter‐directed MRA and fast interactive coronary MRA, one needs to acquire successive images in a relatively short time.^[^ ^19^ ^]^ In such cases, we define the SI efficiency *SI*_efficiency_ as the signal‐to‐noise ratio (SNR) per square root scan time, which can be given by:

(7) $$SI_{\mathrm{efficiency}}\;\propto \;\frac{\left(1-e^{-\mathrm{TR}\left(R_{10}+r_{1}C\right)}\right)\sin\left(\mathrm{FA}\right)}{\left(1-\cos\left(\mathrm{FA}\right)e^{-\mathrm{TR}\left(R_{10}+r_{1}C\right)}\right)\sqrt{\mathrm{TR}}}e^{-\mathrm{TE}\left(R_{20}+r_{2}C\right)}$$

With the optimized values of TE and FA, Equation (7) can be rewritten as:

(8) $${\mathrm{SI}}_{\mathrm{efficiency}}\;\propto \;\sqrt{\tan h\frac{\left(R_{10}+r_{1}C\right)\mathrm{TR}}{2}}\frac{{e^{-{\mathrm{TE}}_{\min}}}^{\left(R_{20}+r_{2}C\right)}}{\sqrt{\mathrm{TR}}}$$

The derivative of Equation (8) with respect to TR is strictly negative with the condition *R*_1_TR < sinh(*R*_1_TR). Studies have shown that the condition is always true for all feasible values of *R*_1_ and TR, which suggests that Equation (8) is monotonically increasing with decreasing TR. Therefore, the optimized TR with the best SI efficiency can be governed by:

(9) $$TR_{\mathrm{optimal}}=TR_{\min}$$

It is noted that when FA = 90°, Equation (1) describes the SI in the FSE sequence and the mathematical optimization model is detailed in Equations S1–S6 in the Supporting Information.

### Materials and Characterization

The hydrophilic Fe_3_O_4_ nanoparticles from Suzhou XinYing Bio‐Medical Technology Co., Ltd. and the Gd‐DTPA from Bayer HealthCare were used as PUSIONPs and GBCAs, respectively in this work. The PUSIONPs used in this study were synthesized through the thermal decomposition of iron organic precursor and produced through an advanced flow synthesis method developed in the previous work.^[^ ^20^ ^]^ High‐temperature thermal decomposition synthesis ensured uniform particle size distribution, particularly for particles smaller than 5 nm, which is very important for achieving excellent relaxation properties. The diphosphonate methoxy polyethylene glycol (DP‐PEG) was used to modify the surface for better colloidal stability, biocompatibility, and prolonged blood residence time. All these facts make the current PUSIONPs significantly different from the conventional superparamagnetic iron oxide nanoparticles (SPIOs) and ultrasmall superparamagnetic iron oxide nanoparticles (USPIOs). The size of the Fe_3_O_4_ nanoparticles was characterized by a transmission electron microscope (TEM, FEI Tecnai G2 F20, FEI Inc., Washington, DC, USA) with an acceleration voltage of 200 kV. The hydrodynamic diameter of the nanoparticles was measured by using a Malvern Zetasizer (Nano ZS 90, Malvern Ltd., Malvern, UK) equipped with a solid‐state He–Ne laser (*λ* = 633 nm).

### Relaxivity Measurements

The relaxivity measurements were performed on a 3T animal MRI system (MR Solutions Ltd., Guildford, Surrey, UK) with 65 mm diameter quadrature bird cage coil and the gradient strength of 585 mT m^−1^. The inversion recovery fast low‐angle shoot (IR FLASH) sequence was used to measure the longitudinal relaxation time, and the sequence parameters were set as: TR = 12 ms, TE = 6 ms, FA = 8°, image size = 256 × 128, field of view (FOV) = 30 mm × 30 mm, inversion time range = 74–6170 ms and slice thickness = 2 mm. The multiecho multislice (MEMS) images were acquired to measure the transverse relaxation time with the sequence parameters of TR = 1400 ms, TE range = 15–450 ms, FA = 90°, image size = 256 × 192, FOV = 30 mm × 30 mm and slice thickness = 2 mm. The *T*_1_ and *T*_2_ mapping were obtained from the scanner to calculate *R*_1_ and *R*_2_ values, which were then used to determine the relaxivity values (*r*_1_ and *r*_2_) by performing linear regression of *R*_1_ and *R*_2_ values against contrast agent concentration.

### Phantom Experiments

To validate the mathematical optimization model, phantoms with serially diluted PUSIONPs and GBCAs were constructed with the following concentrations: 0.01 × 10^−3^ m, 0.02 × 10^−3^ m, 0.04 × 10^−3^ m, 0.05 × 10^−3^ m, 0.06 × 10^−3^ m, 0.08 × 10^−3^ m, 0.10 × 10^−3^ m, 0.20 × 10^−3^ m, 0.30 × 10^−3^ m, 0.40 × 10^−3^ m, 0.50 × 10^−3^ m, 1 × 10^−3^ m, 2 × 10^−3^ m and 3 × 10^−3^ m. The phantoms with pure water were also constructed for comparison. The FSE and 3D FLASH sequences were used to scan the phantoms. A TR range of 100–8000 ms was tested for the FSE sequence with the following parameters: TE = 11 ms, FA = 90°, image size = 256 × 252, FOV = 30 mm × 30 mm and slice thickness = 2 mm. A FA range of 10–60° was used for the 3D FLASH sequence with the parameters of TR = 10 ms, TE = 4 ms, image size = 192 × 154, FOV = 30 mm × 30 mm and slice thickness = 1 mm. The region of interest (ROI) of each phantom was selected to measure the SI, and the data analysis was performed in MATLAB (R2018b, MathWorks, Natick, MA, USA).

### In Vivo Experiments

Female rats (Sprague Dawley, 9–10 weeks old, weight 250–300 g) were used for the pharmacokinetic (*N* = 3 biologically independent animals) and MRA study. All the rats were anesthetized with 3% isoflurane through a nose cone during the MRI scan. All the animal studies were performed according to the guidelines approved by the Ethics and Animal Care Committee of Soochow University.

The iron doses of 0.10, 0.05, and 0.03 mmol kg^−1^ were injected through the tail vein. A Gd dose of 0.10 mmol kg^−1^ was also injected for comparison. Blood samples of each rat were collected at 1, 15, 30, 60, 120, 240, 360, 480, and 720 min after the intravascular injection. To eliminate the interference of endogenous iron in the blood, the plasma containing Fe_3_O_4_ nanoparticles was obtained through ultracentrifugation. Then, the Fe or Gd content of each plasma/blood sample was quantified using inductively coupled‐plasma atomic emission spectroscopy (ICP‐AES) (Agilent 7900, Agilent Technologies, Santa Clara, California, USA). Based on the blood sample measurement, a standard two‐compartment pharmacokinetic model was used to estimate the dynamic intravascular concentrations at any time point after the intravascular injection.^[^ ^21^ ^]^ CE‐MRA images with a Gd dose of 0.10 mmol kg^−1^ and iron doses of 0.10, 0.05, and 0.03 mmol kg^−1^ were acquired using the 3D FLASH sequence (TR = 10 ms, TE = 4 ms, FA = 30°, image size = 192 × 154, FOV = 70 mm × 70 mm, and slice thickness = 1 mm) at 1, 15, 30, 60, 120, 240, 360, 480 and 720 min after the injection. The SI of Gd‐ and iron‐enhanced MRA images at each time point were compared. To investigate the effect of dynamic intravascular concentrations after the PUSIONPs injection, the Ernst angle at each time point was calculated. CE‐MRA images with iron doses of 0.10, 0.05, and 0.03 mmol kg^−1^ were acquired with the calculated Ernst angle. In addition, CE‐MRA images were also acquired with a range of FA values (10–60°) and TR values (10–40 ms) for comparison. All CE‐MRA images were reconstructed by the MIP method.

### Statistical Analysis

Data were presented as the mean ± standard derivation (SD) (*n* = 3) and were carried out using Excel Software.

## Conflict of Interest

The authors declare no conflict of interest.

## Author Contributions

C.L. and S.S.S. contributed equally to this work. Conceptualization: C.L., S.S.S. and J.F.Z.; Investigation: L.C., M.J.A. and D.D.K. Methodology: C.L., K.L., H.Z.W. and N.W.; Writing‐original draft: C.L., S.S.S. and C.Y.L.; Writing‐review and editing: M.Y.G., Y.G., and F.L.; Supervision: M.Y.G., S.S.S., and J.F.Z.; Funding acquisition: M.Y.G., S.S.S., J.F.Z., and L.C. All authors contributed to the general discussion. The final version of the manuscript was approved by all authors.

## Supporting information

Supporting Information

## Data Availability

The data that support the findings of this study are available from the corresponding author upon reasonable request.
