# Supplementary material for: Using Adaptive Imaging Parameters to Improve PEGylated Ultrasmall Iron Oxide Nanoparticles‐Enhanced Magnetic Resonance Angiography
Source: Adv Sci (Weinh). 2024 Aug 20;11(39):2405719. doi: 10.1002/advs.202405719 (PMC11497041; doi:10.1002/advs.202405719)
Supplement: Supplementary file 1 — Supporting Information [file ADVS-11-2405719-s001.pdf]

ADVANCED SCIENCE
Open Access

Supporting Information

for *Adv. Sci.*, DOI 10.1002/advs.202405719

Using Adaptive Imaging Parameters to Improve PEGylated Ultrasmall Iron Oxide Nanoparticles-Enhanced Magnetic Resonance Angiography

Cang Li, Shanshan Shan, Lei Chen, Mohammad Javad Afshari, Hongzhao Wang, Kuan Lu, Dandan Kou, Ning Wang, Yang Gao, Chunyi Liu, Jianfeng Zeng, Feng Liu and Mingyuan Gao**

Supporting Information

Using Adaptive Imaging Parameters to Improve PEGylated Ultrasmall Iron Oxide Nanoparticles-Enhanced Magnetic Resonance Angiography

Cang Li, Shanshan Shan, Lei Chen, Mohammad Javad Afshari, Hongzhao Wang, Kuan Lu, Dandan Kou, Ning Wang, Yang Gao, Chunyi Liu, Jianfeng Zeng, Feng Liu, and Mingyuan Gao**

1. Supplementary Methods

1.1 The Mathematical Optimization Model

When FA = 90° in the Equation (1), the following equations were used to measure the signal intensity (SI) in the fast spin echo (FSE) sequence with contrast agent:

$$\mathrm{SI} \propto (1-\mathrm{e}^{-TR(R_{10}+r_1C)})\mathrm{e}^{-\mathrm{TE}(R_{20}+r_2C)} \tag{S1}$$

Equation (S1) indicates that SI varies monotonically with TE. Therefore, optimal SI occurs at the minimum TE achievable within hardware/software constraints:

$$\mathrm{TE}_{\mathrm{optimal}} = \mathrm{TE}_{\mathrm{min}} \tag{S2}$$

The next variable to be optimized is the concentration of contrast agent. By taking the derivative of Equation (S1) with respect to C:

$$\frac{\partial \mathrm{SI}}{\partial C} = \mathrm{e}^{-\mathrm{TE}_{\mathrm{min}}(R_{20}+r_2C)}\left(\mathrm{e}^{-\mathrm{TR}(R_{10}+r_1C)}(r_2\mathrm{TE}_{\mathrm{min}}+r_1\mathrm{TR})-r_2\mathrm{TE}_{\mathrm{min}}\right) \tag{S3}$$

Equation (S3) is equated to zero ($\partial\mathrm{SI}/\partial C = 0$) and solved for the optimal concentration:

$$C_{\mathrm{optimal}} = \frac{\log(1+D)-R_{10}\mathrm{TR}}{r_1\mathrm{TR}} \tag{S4}$$

Where $D = (r_1\mathrm{TR})/(2r_2\mathrm{TE}_{\mathrm{min}})$ in Equation (S4). By taking the derivative of Equation (S1) with respective to TR:

$$\frac{\partial \mathrm{SI}}{\partial \mathrm{TR}} = \mathrm{e}^{-\mathrm{TE}_{\mathrm{min}}(R_{20}+r_2C)}\mathrm{e}^{-\mathrm{TR}(R_{10}+r_1C)}(R_{10}+r_1C) \tag{S5}$$

Mathematically, as $\partial\mathrm{SI}/\partial\mathrm{TR} > 0$ and increasing TR will increase the maximal SI.

Substituting Equation (S4) in Equation (S1) resulted in:

$$\mathrm{SI}_{\mathrm{max}} \propto -\mathrm{e}^{\left(-\mathrm{TE}_{\mathrm{min}}\left(R_{20}-\frac{r_2\left(R_{10}-\frac{\log(1+D)}{\mathrm{TR}}\right)}{r_1}\right)\right)\left(\frac{1}{1+D}+1\right)} \tag{S6}$$

The result indicates that for any contrast agent with known relaxivity properties (r_1, r_2),

the corresponding C (C_{optimal}) and the theoretical maximum SI (SI_{max}) could be predicted from Equation (S4) and Equation (S6), respectively.

1.2 The Two-Compartment Pharmacokinetic Model

A standard two-compartment pharmacokinetic model was used to estimate the dynamic intravascular concentrations. The closed-form solution to this model is the well-described bi-exponential equation:

$$C(t) = A\mathrm{e}^{-\alpha t} + B\mathrm{e}^{-\beta t} + c \tag{S7}$$

Where constants A and α describe the distribution phase; B and β describe the clearance phase.

2. Supplementary Results

Table S1. The fitted parameters from the two-compartment pharmacokinetic model of intravascular concentrations after the injection of Gd-DTPA and Fe_3O_4 nanoparticles in rats.

	0.10 mmol kg^{-1} Gd-DTPA	0.10 mmol kg^{-1} Fe_3O_4 nanoparticles	0.05 mmol kg^{-1} Fe_3O_4 nanoparticles	0.03 mmol kg^{-1} Fe_3O_4 nanoparticles
A	7.87	0.70	0.22	1.24
B	-0.60	-0.07	-1.51	-0.007
α	0.20	3.99	2.19	0.17
β	-0.03	-0.002	-0.005	-0.007
c	0	0	0	0.10
$t_{1/2}$, α	0.76	9.6	0.50	0.71
$t_{1/2}$, β	24	288	154	100

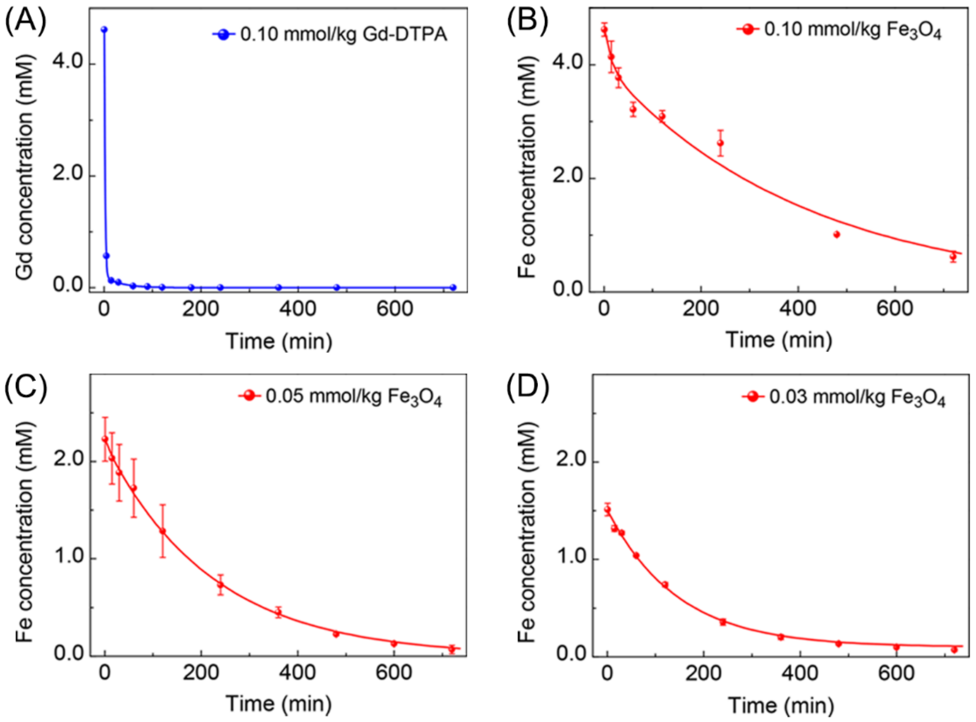

Figure S1. The intravascular concentrations after the injection of A) 0.10 mmol kg^{-1} Gd-DTPA, B) 0.10 mmol kg^{-1}, C) 0.05 mmol kg^{-1}, and D) 0.03 mmol kg^{-1} Fe_3O_4 nanoparticles in rats fitted with the two-compartment pharmacokinetic model.

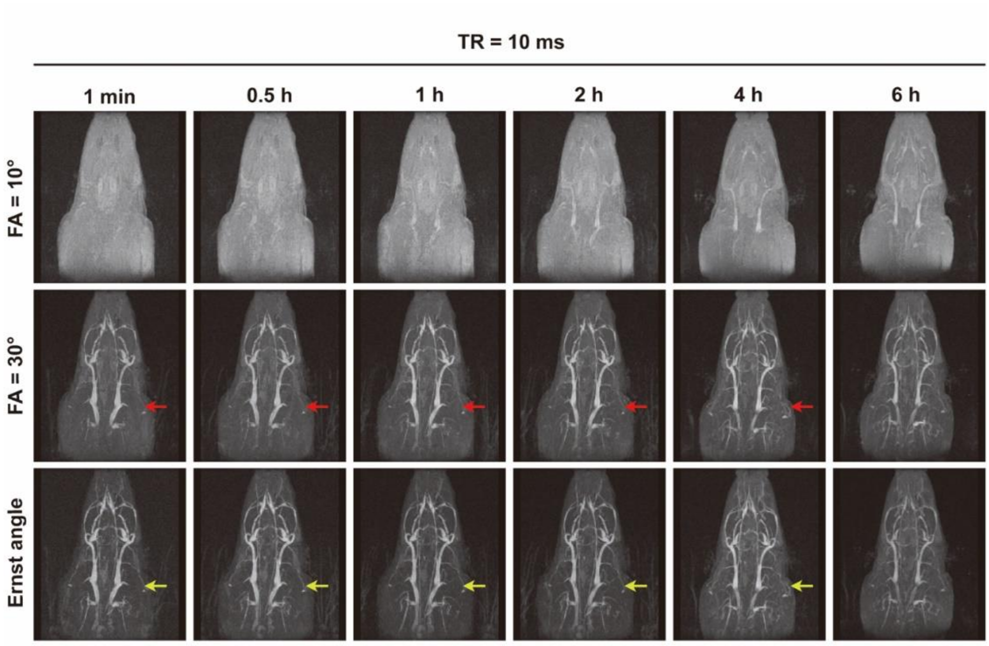

Figure S2. The 3D maximum intensity projection (MIP) reconstructions of rat CE-MRA images within 6 h after the injection of 0.10 mmol kg^{-1} Fe_3O_4 nanoparticles acquired with the fixed FA values (10° and 30°) and the adaptive Ernst angle values, respectively at TR = 10 ms and TE = 4 ms.

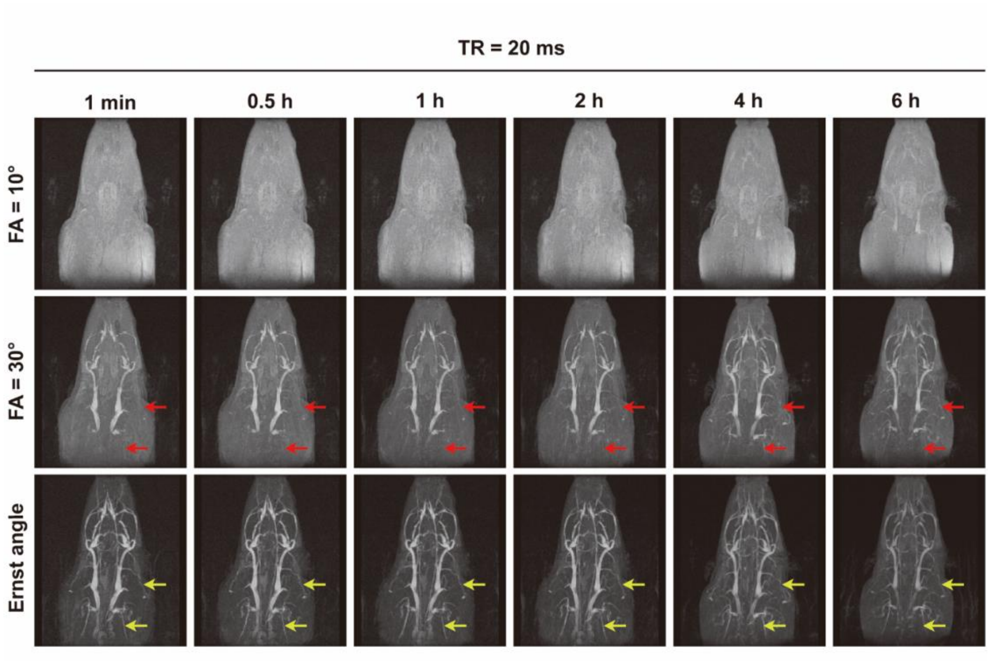

Figure S3. The 3D MIP reconstructions of rat CE-MRA images within 6 h after the injection of 0.10 mmol kg^{-1} Fe_3O_4 nanoparticles acquired with the fixed FA values (10° and 30°) and the adaptive Ernst angle values, respectively at TR = 20 ms and TE = 4 ms.

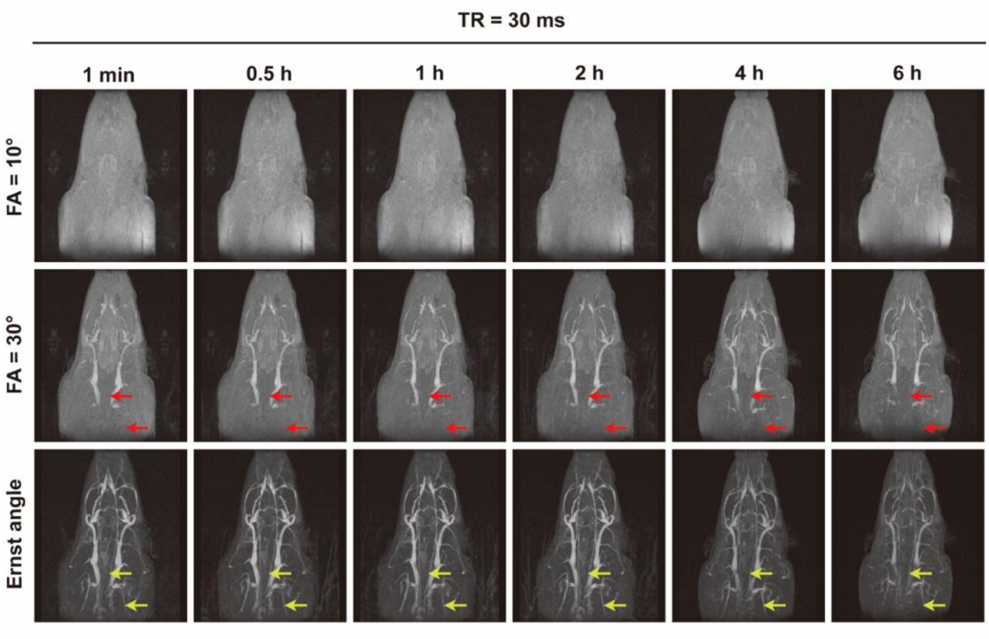

Figure S4. The 3D MIP reconstructions of rat CE-MRA images within 6 h after the injection of 0.10 mmol kg^{-1} Fe_3O_4 nanoparticles acquired with the fixed FA values (10° and 30°) and the adaptive Ernst angle values, respectively at TR = 30 ms and TE = 4 ms.

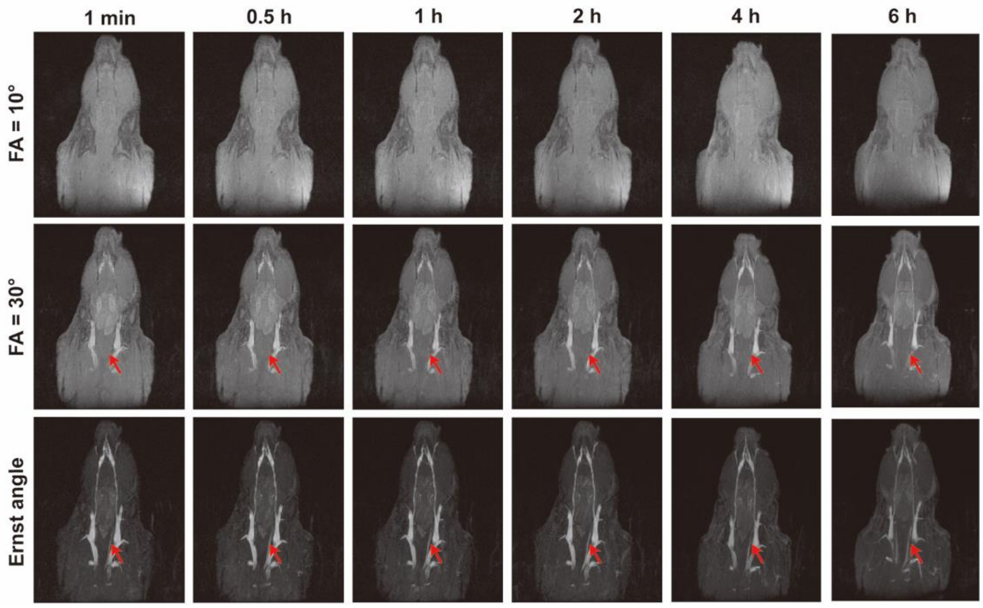

Figure S5. The 3D thin MIP reconstructions of rat CE-MRA images within 6 h after the injection of 0.10 mmol kg^{-1} Fe_3O_4 nanoparticles acquired with the fixed FA values (10° and 30°, the top two rows) and the adaptive Ernst angle values (the third row), respectively at TR = 40 ms and TE = 4 ms. The red arrows denote the right carotid artery.
